# Divergent behavioural responses of gypsy moth (*Lymantria dispar*) caterpillars from three different subspecies to potential host trees

**DOI:** 10.1038/s41598-019-45201-3

**Published:** 2019-06-20

**Authors:** Andrea Clavijo McCormick, Luca Arrigo, Helen Eggenberger, Mark C. Mescher, Consuelo M. De Moraes

**Affiliations:** 1 Massey University, School of Agriculture and Environment, Private Bag 11222, 4442 Palmerston North, New Zealand; 2 Department of Environmental Systems Science, ETH Zürich, Schmelzbergstrasse 9, 8092 Zürich, Switzerland

**Keywords:** Plant sciences, Ecology, Entomology

## Abstract

Almost all previous work on host-plant selection by insect herbivores has focused on adult behaviour; however, immature life stages can also play an active role in host discrimination. The important forest pest *Lymantria dispar* (gypsy moth) has three recognised subspecies: the European, Asian, and Japanese gypsy moth. Unlike the other two subspecies, the European subspecies is characterised by a loss of female flight ability, which might impose a selective pressure on larvae to actively engage in host-plant selection. We therefore explored the interactions of early-instar larvae from laboratory colonies of each subspecies with four potential hosts of differing quality: oak, beech, maple, and pine—measuring larval survival and performance, feeding preferences, responses to host-derived odour cues, and the propensity to disperse from hosts via ballooning. Compared to larvae from the Asian and Japanese subspecies, larvae from the (American-originated) European gypsy moth colony exhibited (i) significantly lower survival on the poorest quality host (pine), (ii) an ability to discriminate among hosts via olfactory cues; and (iii) higher propensity to disperse from sub-optimal hosts. These results are consistent with the hypothesis that larvae from flightless female European Gypsy moth subspecies play a more active role in host-plant selection.

## Introduction

The behavioural and sensory mechanisms by which insect herbivores locate and select among potential host plants have been extensively documented^1–6^. However, most of this work has focused on the selection of plants for feeding or egg-laying by adult insects, while relatively little is known about the role of immature life stages, such as Lepidopteran larvae, in host-plant selection and discrimination^7–9^. Yet, access to suitable host plants is critical for neonate and early instar larvae, whose survival can be compromised by feeding upon an unsuitable host^8^. Furthermore, active discrimination by larvae may be particularly important in polyphagous species, where the oviposition preferences of adult females are not always correlated with offspring performance^10^. In addition to having relevance for the ecology and evolution of plant-herbivore interactions, improved understanding of the role of immature insects in host selection may inform efforts to control damaging insect pests, particularly in the case of biological invaders and species with broad geographic ranges.

The gypsy moth (*Lymantria dispar*) is an important forest pest worldwide with three recognised subspecies. The European gypsy moth (EGM) is distributed across most of temperate Europe, from Portugal to the Ural Mountains, and in the late 19th century was introduced into North America, where it soon became a major forest pest^11–13^. The Asian gypsy moth (AGM) occurs east of the Ural Mountains (mainly in Russia, China, Japan, and Korea), and the Japanese gypsy moth (JGM) occurs on all of the major Japanese islands^11,12,14^. Goldschmidt^15^ first documented morphological and developmental variation among gypsy moth subspecies from Europe, East Asia and Japan, and posited that this variation might have a genetic basis. Later studies confirmed the genetic divergence of the subspecies and revealed reduced levels of genetic variability in North American subspecies relative to those from Europe, Asia, and Japan^16^. Recent studies^17^ investigating microsatellite loci and mitochondrial DNA sequences for 1738 individuals across the globe, found three genetic clusters corresponding to the three named subspecies, with the North American subspecies representing a separate cluster, likely due to the population bottleneck that accompanied the introduction of the gypsy moth to the US.

Differences among subspecies, particularly in the mechanisms by which they select host trees, may have important ecological implications, including their ability to colonize and spread within new geographic areas. The complete loss of flight ability in females is a key morphological difference that sets EGM apart from the other two subspecies^11,12^. Females of AGM and JGM are flight capable and have comparatively larger wings, well-developed flight muscles, and lower abdominal mass^18^ compared to EGM females. The inability to fly prevents EGM females from actively selecting suitable hosts for their offspring^11,12^, which, in turn, might be expected to create selection pressure favouring a more active role of early larval stages in host selection.

Differences in ecological factors such as climate, host availability, and the presence of natural enemies across the geographic ranges of different subspecies may also act as selective pressures influencing the evolution of host selection mechanisms. Therefore, we compared the host selection behaviour of the three recognised subspecies using lab colonies originating from different geographical locations.

The American EGM colony used in the current study originated from New Jersey (U.S). This region is characterized by mild and humid winters and a high diversity of potential host-plants, dominated by 20 different Oak species^19,20^. Likely as a result of its recent introduction, the EGM has few natural enemies in the North American range^21,22^.

The JGM colony originated from Southern Japan. This region typically experiences short and mild winters; however, like many island ecosystems, the range of the JGM is characterized by a high diversity of endemic plant species, including the Japanese Oaks *Quercus acuta, Q. stenophylla* and *Q. hondae* and the conifers *Pinus thunbergii* and *Cryptomeria japonica* (Japanese black pine and Japanese cedar, respectively)^23,24^. Gypsy moths in this range also have many natural enemies, including the tachinid fly *Exorista japonica* and the fungal pathogen *Entomophaga maimaiga*^25–27^.

The AGM colony was started using egg masses from the Primorskiy-Krai region in South-East Russia. This region typically experiences warm, humid and rainy summers and cold dry winters, with temperatures dropping below −20 °C due to the influence of the Siberian High^28^. There are two main vegetation zones dominated by conifers, the western Okhost dark conifer areas and the Manchurian-North Japanese mixed (broadleaf + conifer) forest areas in the coastal regions^29^. The predominant forest trees are conifers *Picea ajanensis, Pinus koraiensis, and Pinus pumila*, and deciduous trees *Quercus mongolica* and *Betula platyphylla*^29^. Only a few natural enemies of gypsy moths have been reported in this range, with entomopathogens among the most important, and there are low rates of parasitism by hymenopteran and dipteran parasitoids^30^.

We recently investigated chemosensory differences among larvae of the three subspecies^31^, but no previous work has examined subspecies variation in larval host-finding behaviour. We hypothesize that larvae from the three subspecies may exhibit differences in host preferences and host-selection behaviour as a result of differences in female flight capacity and ecological factors associated with their distribution ranges (e.g., climate, presence of natural enemies, and available hosts), the current study explored responses of early-instar larvae from lab colonies of the three recognised subspecies to host plants of differing quality. Throughout the manuscript we refer to the different lab colonies as subspecies, abbreviating them as EGM, AGM and JGM. Our main goals were to explore: (i) whether differences exist in the survival and growth of gypsy moth larvae from these subspecies on different host plants; (ii) whether the subspecies exhibit different behavioural responses to potential hosts, and (iii) whether larval preferences align with host-plant quality. To gain insight into these questions we tested larval performance and behavioural responses to four different woody plants from representative genera that are distributed throughout the northern hemisphere and that were expected, based on previous literature, to differ in host quality for *L. dispar*^32–36^. The selected tree species (in descending order of expected quality) were: *Quercus robur* (pedunculate oak), *Fagus sylvatica* (common beech), *Acer campestre* (field maple) and *Pinus sylvestris* (Scots pine). We explored the interactions of gypsy moth larvae from each subspecies with these potential hosts through a series of bioassays, including performance assays, dispersal (ballooning) assays, Y-tube olfactometer assays, and multiple-choice feeding preference assays. This is the first report comparing behavioural responses of the larvae from the three *L*. d*ispar* subspecies and thereby provides new insights into the ecology of this important pest species and, more generally, into the process of host selection by early instar Lepidopteran larvae.

## Results

### Survival and performance assays

**Figure 1:**
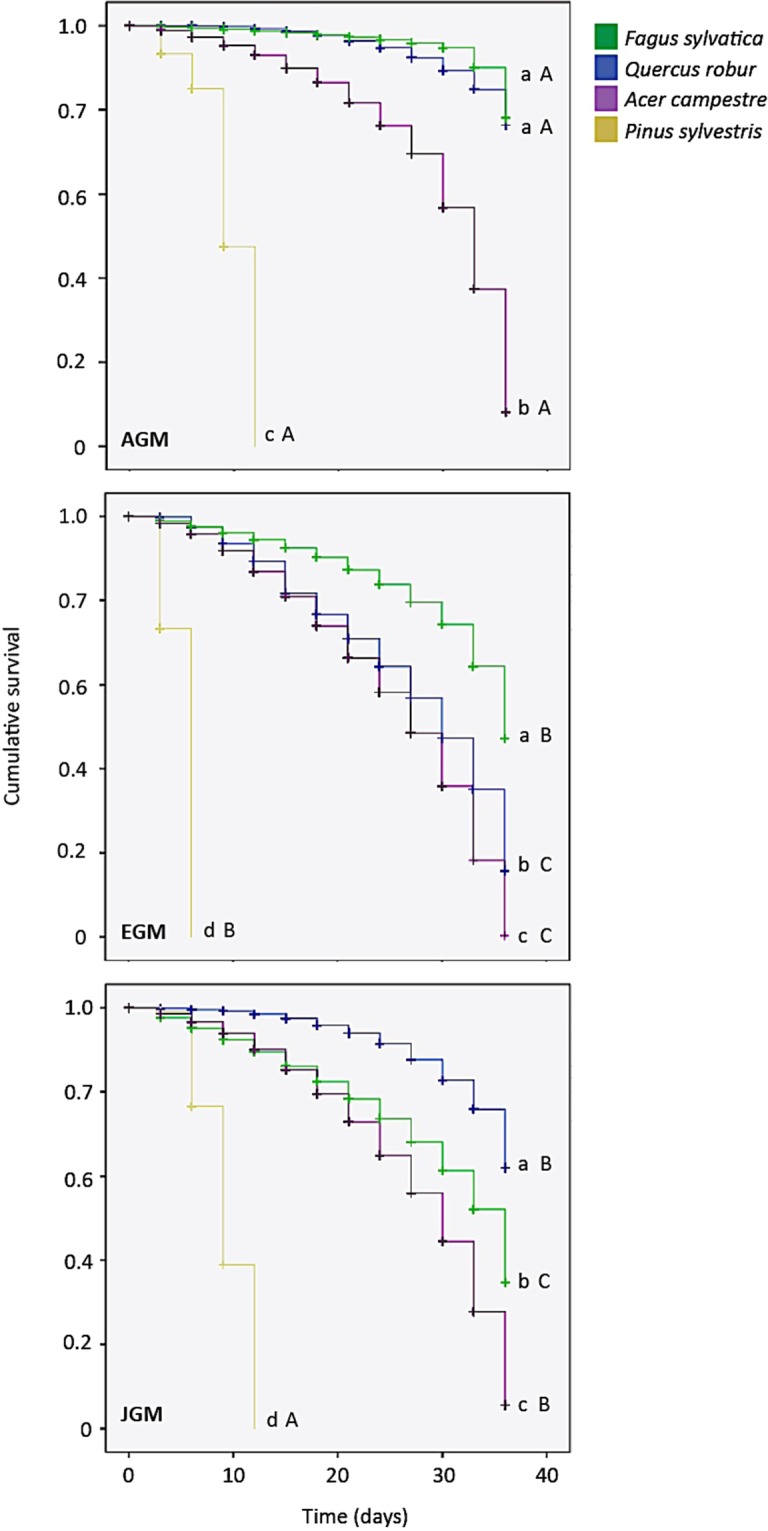
Cumulative survival of larvae from three gypsy moth subspecies on four woody plant species. AGM = Asian gypsy moth (*Lymantia dispar asiatica*), EGM = European gyspsy moth (*L. dispar dispar*), JGM = Japanese gypsy moth (*L. dispar japonica*). Letters indicate significant differences after a Breslow (Generalized Wilcoxon) test followed by pairwise comparisons. Lowercase letters depict significant differences (P < 0.05) in survival within the subspecies; uppercase letters indicate significant differences among subspecies.

Assays of larval survival and growth on the different hosts were conducted by placing freshly hatched larvae from each subspecies into petri dishes containing fresh foliage from one of the focal tree species. Analysis of the survival distributions revealed significant differences among the three subspecies (Table S1). JGM larvae had significantly higher survival on oak (*Q. robur*) than on the other species, while EGM larvae showed the highest survival rates on beech (*F. sylvatica*), and AGM larvae survived equally well on both oak and beech. For all three subspecies, survival was lowest on pine (*P. sylvestris*), yet both AGM and JGM had significantly higher survival on pine than EGM (Fig. 1). Survival rates on maple (*A. campestre*) were intermediate between oak/beech and pine in all cases. Overall, AGM larvae had the highest survival rates on all plant species used in this experiment.

A two-way ANOVA revealed a significant effect of subspecies (F = 4.71, df = 2, p = 0.014), host plant (F = 48.5, df = 2, p < 0.001), and their interaction (F = 9.75, df = 4, p < 0.04) on the weight of second instar larvae (Table 1), with larvae of all subspecies having higher weights following feeding on beech or oak than on maple (pine could not be quantified due to low larval survival rates). AGM had the lowest weight and JGM the highest (and these were significantly different), with EGM having intermediate values not different from those of AGM or JGM (Table 1).

**Table 1:** Mean weight of second instar larvae, standard error of the mean (SEM) and results of a post-hoc test (Tukey) after a two-way ANOVA, estimating the effects of subspecies, plant species, and their interaction on the weight of second instar larvae.

*Subspecies*	*plant species*	*Mean weight (mg)*	±*SEM*	*Tukey*
AGM^1^	*Acer campestre*	1.450	0.200	
	*Fagus sylvatica*	3.787	0.147	
	*Quercus robur*	2.908	0.392	
	**Total**	**2.715**	**0.275**	**a**
EGM^1^	*Acer campestre*	1.220	0.302	
	*Fagus sylvatica*	5.198	0.263	
	*Quercus robur*	3.437	0.934	
	**Total**	**3.400**	**0.536**	**a,b**
JGM^1^	*Acer campestre*	1.625	0.153	
	*Fagus sylvatica*	4.550	0.611	
	*Quercus robur*	4.877	0.564	
	**Total**	**3.684**	**0.443**	**b**
**Total**	*Acer campestre*	**1.444**	**0.124**	**A**
	*Fagus sylvatica*	**4.512**	**0.255**	**B**
	*Quercus robur*	**3.801**	**0.387**	**B**

Differing lowercase letters indicate significant differences between subspecies and uppercase letters indicate significant differences between plant species.

^1^AGM = Asian gypsy moth, EGM = European gypsy moth, JGM = Japanese gypsy moth.

### Ballooning assays

**Figure 2:**
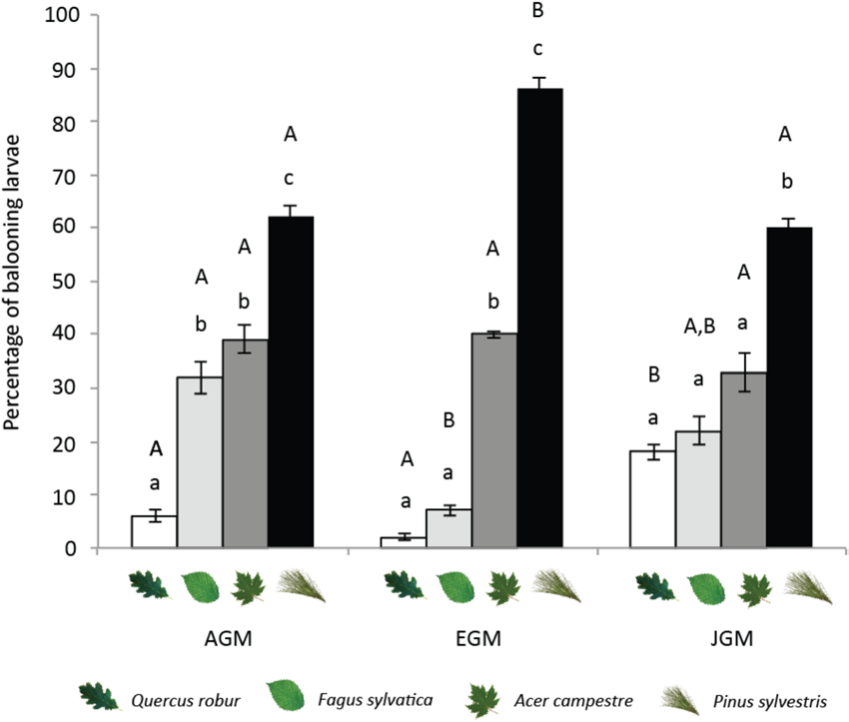
Percentage of ballooning larvae of three gypsy moth subspecies on four woody plant species. AGM = Asian gypsy moth, EGM = European gypsy moth, JGM = Japanese gypsy moth. Letters indicate significant differences after parametric or non-parametric ANOVA, followed by post-hoc comparisons (Tukey or Mann-Whitney). Lowercase letters depict differences (P < 0.05) in the percentage of ballooning within the subspecies; uppercase letters indicate significant differences among subspecies.

To assess the propensity of early instar larvae to disperse from unfavourable hosts, we measured the rates of ballooning (a means of dispersal)^37^ on the four tree species. The three subspecies showed significant differences in their ballooning rates on different host-plants (Fig. 2; statistical details can be found in Table S2**)**. In all cases, significantly more larvae ballooned when placed on pine compared to other tree species; however, EGM larvae ballooned significantly more often from pine than AGM or EGM larvae. The tendency to balloon from the other tree species varied among subspecies (Fig. 2).

### Multiple-choice feeding assays

**Figure 3:**
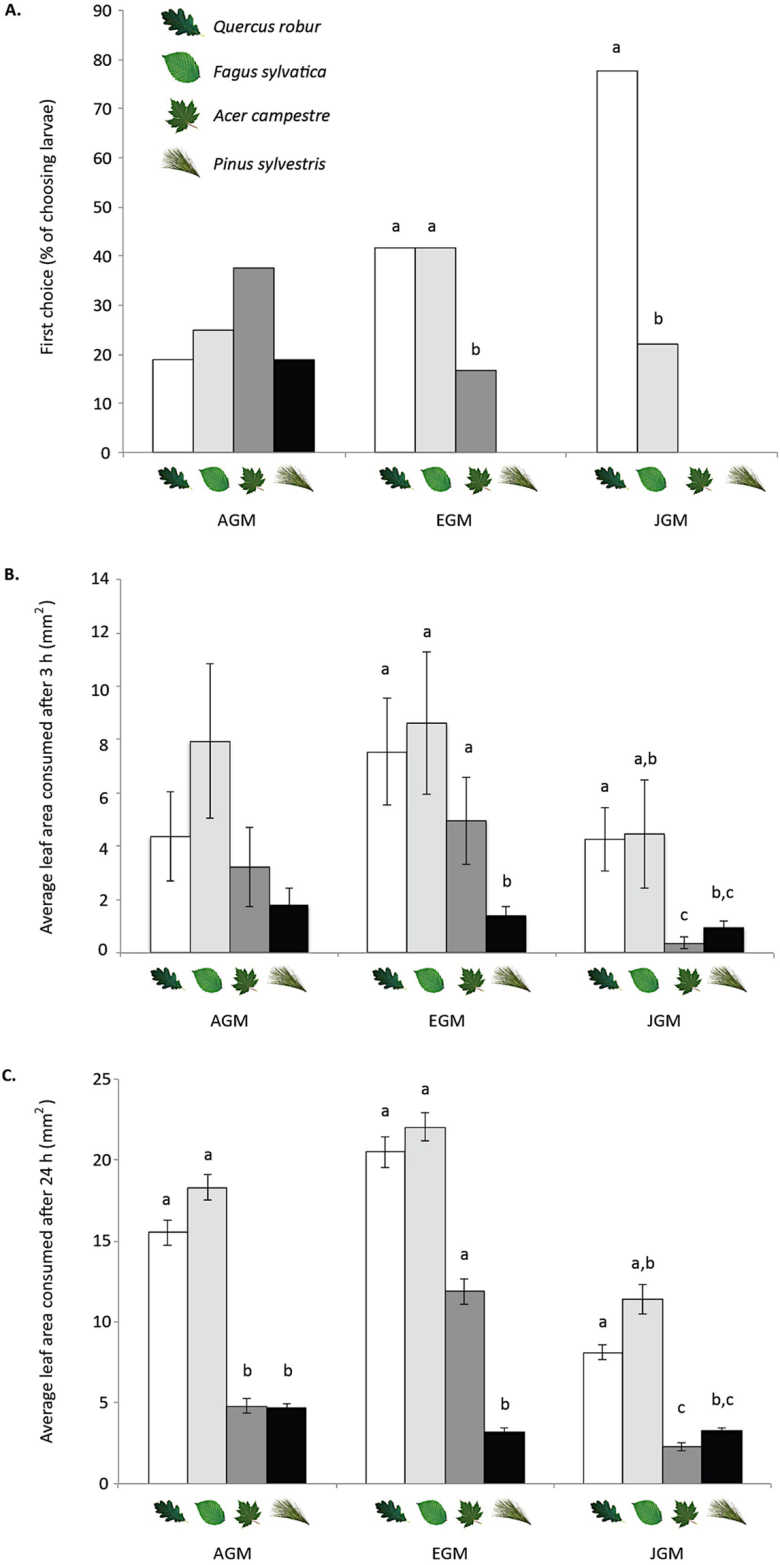
Results of multiple-choice feeding assays for three subspecies of the gypsy moth, *Lymantria dispar*. AGM = Asian gypsy moth, EGM = European gypsy moth, JGM = Japanese gypsy moth. (**A)** First choice, (**B**) Average leaf area consumed after 3 h, (**C**) Average leaf area consumed after 24 h. Lowercase letters depict significant differences (P < 0.05) in first choice and leaf area consumed within the subspecies. (**A**) Chi^2^ tests followed by pairwise comparisons, and (**B**,**C**) Friedman tests followed by Wilcoxon signed-rank comparisons.

To directly assess larval preference for the different potential host plants we presented second instar larvae with leaf discs from the four tree species and monitored feeding rates. Initial larval feeding preference varied significantly for EGM and JGM (Chi^2^ = 49.99, P < 0.001 and Chi^2^ = 161.7, P < 0.001 respectively). EGM larvae significantly preferred beech and oak to maple, and never selected pine as their first choice, while JGM larvae were more selective, preferring oak or beech and never choosing maple or pine (Fig. 3A). In contrast, AGM larvae exhibited no significant initial preference among hosts (Fig. 3A).

After 3 hrs, we found significant variation in feeding preferences for EGM (Chi^2^ = 9.278, P = 0.026) and JGM (Chi^2^ = 9.922, P = 0.019), but not AGM (Fig. 3B). Posterior pairwise analyses using a Wilcoxon signed-ranked test showed that EGM larvae consumed significantly more beech, oak, and maple than pine. By contrast, JGM larvae consumed significantly less maple and pine than the other host trees (Fig. 3B).

After 24 hours, there were significant differences among the four trees species in the leaf areas consumed by all three moth subspecies (Fig. 3C) (AGM Chi^2^ = 8.456, P = 0.037; EGM Chi^2^ = 18.233, P < 0.001; JGM Chi^2^ = 12.94, P = 0.005). All subspecies consumed significantly more oak than either maple or pine at this time point (Fig. 3C).

### Y-tube olfactometer assays

**Figure 4:**
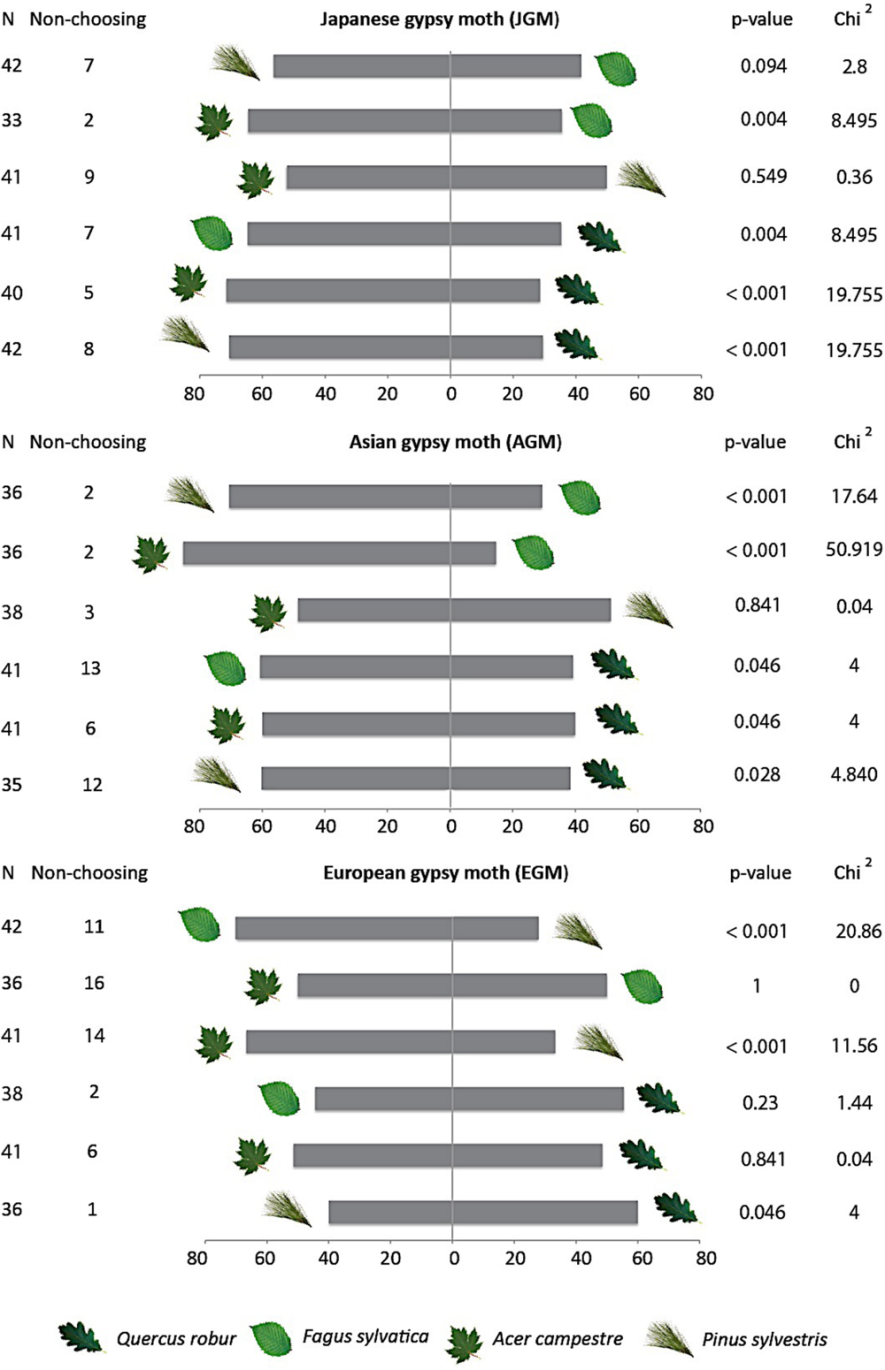
Results of pairwise Y-tube olfactometer tests evaluating the preferences of larvae from three gypsy moth subspecies for plant odours from different host species. AGM = Asian gypsy moth, EGM = European gypsy moth, JGM = Japanese gypsy moth. N = total number of larvae tested, Non-choosing = number of larvae not making a choice. P and Chi^2^ values are given.

To determine larval preferences between the volatile blends of the four tree species, we conducted choice tests with individual larvae in a Y-tube olfactometer using pairwise combinations of all tree species. These assays revealed different patterns in the use of olfactory cues by larvae of the three subspecies. Olfactory preferences did not correlate with the estimates of survival and performance obtained in this study for either AGM or JGM, with both of these subspecies preferring the odours of pine and maple to those of more suitable hosts such as oak or beech (Fig. 4). In contrast, EGM larvae avoided the odour of pine, but did not display clear preferences among oak, beech, or maple (Fig. 4).

### Plant volatiles

**Figure 5:**
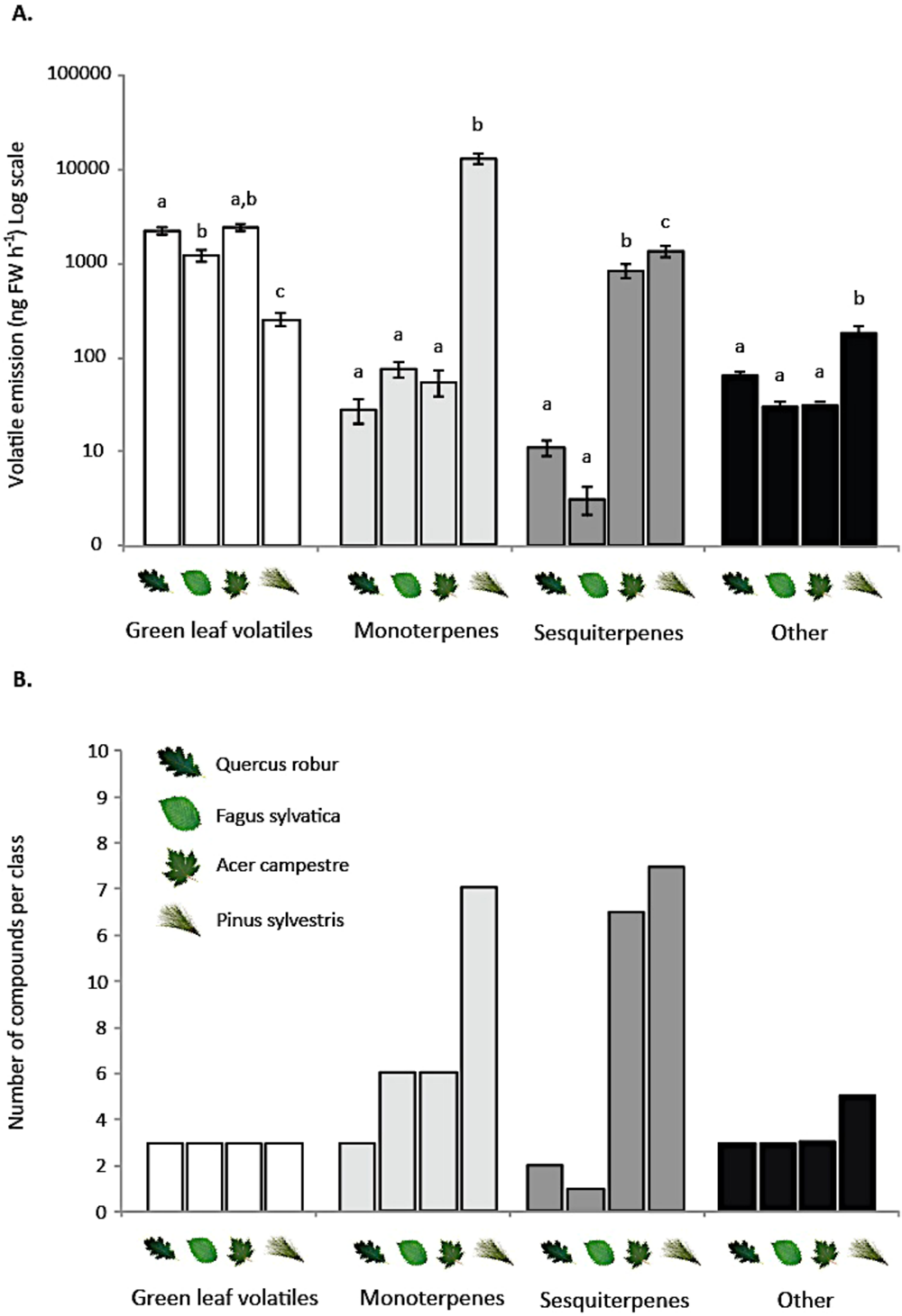
Results from headspace volatile analysis of four gypsy moth host-plant species: pedunculate oak (*Q. robur*), common beech (*F. sylvatica*), field maple (*A. campestre*) and Scots pine (*P. sylvestris*). (**A**) Volatile emission per compound class in ng FW h^−1^ and (**B**) Number of compounds per chemical class. In (**A**). Lowercase letters indicate significant differences P < 0.05.

We documented 11 volatile compounds in the headspace of the pedunculate oak (*Q. robur*), 14 for the common beech (*F. sylvatica*), 25 for field maple (*A. campestre*), and 37 for Scots pine (*P. sylvestris*) (Fig. 5B). Pine saplings emitted the highest total amount of volatile compounds (14332 ± 1814 ng FW h^−1^), with a headspace heavily dominated by monoterpenes. Maple was the second highest emitting plant (3350 ± 396 ng FW h^−1^), followed by oak (2321 ± 238 ng FW h^−1^), whereas beech had the lowest emission rates (1301.9 ± 178.5). Green-leaf volatiles dominated the headspace of maple, oak, and beech (Fig. 5A,B). Pine released significantly less green-leaf volatiles than the other species (ANOVA, F = 97.56, P < 0.001), but considerably more monoterpenes, sesquiterpenes and other compounds (ANOVA, F = 97.56, P < 0.001; F = 44.84, P < 0.001; F = 18.46, P < 0.001 respectively) (Fig. 5A).

## Discussion

Our findings reveal differences in the survival and performance of lab-reared gypsy moth larvae belonging to three different subspecies (EGM, AGM and JGM) on four potential host trees (beech, oak, maple and pine). The relative quality of the four tree species was similar across all three subspecies, with oak and beech being high-quality hosts, pine being consistently the least suitable host, and maple exhibiting intermediate quality. However, EGM larvae had significantly lower survival rates on pine than both AGM and JGM larvae.

Our results are consistent with previous reports for EGM larvae from North America, which showed that oak trees (*Quercus* spp.) are the preferred (optimal) hosts, while pine trees (*Pinus* spp.) are the least preferred (suboptimal) hosts^32–36^. The current results extend these findings, showing that this pattern holds true for different gypsy moth subspecies (EGM, AGM and JGM) and additionally indicate that beech (*Fagus* spp.) can also be a highly suitable host, while maple (*Acer* spp.) has intermediate quality.

The quality of a given plant for polyphagous insects like *L. dispar* can be strongly influenced by plant defensive biochemistry, including the presence or amount of toxins, repellents or deterrents. An extensive review of the literature reveals that potential host plants differ in the composition of their chemical defences and that the presence of alkaloids renders a plant unsuitable for consumption by the EGM^38–41^. Of the four host species tested, maple is well known to possess alkaloids^40^, whereas these are mostly absent from beech and oak^39^. Pine also produces toxic alkaloids^42,43^, but is better known for producing copious amounts of other secondary metabolites (terpenes, resin acids and phenolics) responsible for its allelopathic properties. In addition to alkaloids, other plant-derived compounds have been reported to have antifeedant, repellent and toxic effects on gypsy moth larvae, including diterpene acids^44^, nitrogenous compounds^45–47^, and phenolic glycosides^48,49^. Although not tested directly, our results would be in line with secondary metabolites playing a role in the host-selection process.

The variation in survival and performance of gypsy moth subspecies on different host plants may partly have arisen through natural selection imposed by environmental factors. JGM larvae, for example, might conceivably benefit by accepting suboptimal hosts in order to access an enemy-free space, due to the high abundance and diversity of natural enemies in their range^25,26^. AGM larvae, meanwhile, might face pressure to exploit sub-optimal hosts due to the predominance of conifer species in their distribution range^29^. By contrast, EGM larvae from North America encounter few natural enemies in the introduced range^21,22^ and have access to a wide variety of suitable hosts^19,22^, and so may not have experienced strong selective pressure to use less suitable hosts. Alternatively, the predicted bottle-neck effect associated with colonisation of North America by EGM may have contributed to the spread of EGM subspecies with already low tolerance to plant secondary metabolites.

In terms of host plant acceptance, we found that the propensity of early-instar larvae to balloon from different hosts was quite similar, with all three subspecies ballooning more frequently from pine than from broad-leaved species. Previous studies also found that first-instar gypsy moths attempt dispersal more frequently when exposed to less acceptable foliage^37^. EGM larvae exhibited a greater propensity to disperse from poor hosts via ballooning than AGM or JGM larvae, which reflects the reduced survival on this host and suggests that EGM larvae has additional behavioural and possibly sensory adaptations to avoid unsuitable hosts. It is, however, important to consider that other factors, such as the nutritional experience of the mother and resource provision of eggs, can also influence the tendency of larvae to balloon under field conditions^50,51^.

In addition to differences in survival, performance, and host acceptance, our results document variation among the three subspecies in the sensory mechanisms and associated behaviours employed to select suitable hosts. In Y-tube olfactometer tests, only EGM used olfactory cues to avoid the unsuitable host (pine), while AGM and JGM tended to prefer plants emitting more volatiles regardless of their quality as hosts. In multiple-choice assays, AGM did not display an initial preference for any of the host plants, and only made a choice about which plant to consume after probing the leaves, suggesting that gustatory cues are important for this subspecies. JGM showed an initial feeding preference towards beech and oak, but based on Y-tube olfactometer tests, these preferences are not olfactory, suggesting that either visual or contact cues are involved. After 24 hours, the feeding patterns on the different host species were similar for all subspecies, indicating that different sensory mechanisms lead to the same outcome. It is important to note that the use of cut leaf material in multiple-choice assays may have caused the release of volatile organic compounds that differ from those emitted by undamaged plants, which may have an impact on the assessment of host preference^52^. Nevertheless, these assays indicate that larvae from the three subspecies have different behavioural approaches to host selection.

The observed differences in the use of sensory cues and their associated behaviour could be related to environmental factors, as discussed above. However, we also speculate that the loss of flight ability by EGM females (prior to the introduction of the EGM to America)^11,12^, and subsequent inability of the females to select suitable hosts, may have been an important factor in EGM larvae developing more sophisticated mechanisms of host selection. Interestingly, a recent transcriptomics study exploring the expression of chemosensory-related genes in larval head capsules^31^ found significant differences in the gene expression patterns for these three gypsy moth subspecies, suggesting that differences are not only behavioural but at the perception level as well.

As noted, only EGM larvae were able to discriminate broad-leaved plants from conifers based on olfactory cues. The plant headspace analysis showed that broad-leaf plants (including high- and intermediate-quality hosts) emit higher amounts of green-leaf volatiles relative to pine (the least suitable host), but lower amounts of terpenes. Taking into consideration that green-leaf volatiles are ubiquitous plant compounds^53,54^, and thus good cues for polyphagous herbivores, it is reasonable to speculate that EGM larvae may use these compounds in combination with others to discriminate between suitable and non-suitable hosts (e.g., green leaf volatile to monoterpene ratio). Previous studies indicate that volatiles are used as foraging cues by the larvae of other phytophagous insects^55–60^, and that these insects are able to infer information about plant quality and the presence of potential competitors from herbivore-induced volatiles. However, there is behavioural plasticity in the use of plant volatiles by caterpillars, and previous experience may play an important role determining subsequent responses to these cues^47^.

Finally, we note that the three subspecies used in this study were sourced from laboratory populations, which were originally founded using individuals from distinct geographical areas. The genetic and phenotypic variation encompassed by our lab colonies is therefore not representative of the variation present within the entire geographic range of each subspecies. Furthermore, lab rearing can impose artificial selection on traits, along with other population genetic factors such as founder effects, genetic drift, and inbreeding, which may also cause genotypic and phenotypic differences relative to the source material^61^, potentially including effects on larval behaviour and performance. However, all of our subspecies were obtained from the same laboratory source and were reared under the same conditions, using carefully designed protocols to minimise deleterious genetic effects, so the observed results are unlikely to reflect differences in rearing practices. In any event, it would be highly desirable for future work to examine how larvae from wild populations of the three subspecies—and ideally from geographically diverse populations within each subspecies—select and interact with host trees.

To summarize, the larvae of three subspecies of the gypsy moth display different host-selection strategies, which may have arisen as a result of selection pressures associated with differences in the mother’s flight capacity and environmental factors unique to their respective geographical ranges. Further research is needed to elucidate the specific mechanisms responsible for the observed differences in larval behaviour and to determine how they influence host finding and discrimination in wild populations.

## Methods

### Insects

Gypsy moth larvae originated from laboratory cultures and were kindly provided by Hannah Nadel at the Animal and Plant Health Inspection Service (APHIS) of the U.S. Department of Agriculture (USDA). The EGM (*Lymantria dispar dispar*) laboratory culture was initiated via collections from *L. dispar* subspecies in New Jersey (USA); the JGM culture (*L. dispar japonica*) was initiated via collections from Hoshu, Takizawa, Morika and Nishine, in the Iwate district of Japan; and the AGM culture (*L. dispar asiatica*) was initiated via collections from the Primorskiy Krai ports (Vostochnyy, Slavyanka, Vladivostok, and Nadhodka) in Southeastern Russia.

Experimental insects were provided as egg masses. After hatching, caterpillars were maintained in a climate chamber (24 °C, 16:8 h light:dark photoperiod, 60% Relative Humidity) and were allowed feed *ad libitum* on either plant material (for survival and weight gain assays) or artificial wheat-germ diet (for behavioural assays). Wheat-germ diet was prepared according to the manufacturer’s instructions (MP Biomedicals LLC, Illkirch, France) and was used to avoid inducing feeding preferences for different host trees.

### Plants

For each of the four tree species listed above, saplings with initial heights between 30 and 60 cm were acquired from a nursery (Emme-Forstbaumschulen A.G.; Rapperswil, Switzerland) and used for experiments when they reached a height of 1.00 to 1.20 m. Saplings were initially transplanted to 3.5 L plastic pots with an 8:1:1 mixture of a commercial substrate (Substrat 2, KlasmannDeilmann GmbH, Germany), vermiculite, and perlite and kept in a climate chamber (24 °C, 16:8 h light:dark photoperiod, 60% RH, and 50% light exposure). Trees were watered weekly and fertilized with 35–45 g of long-duration fertilizer (Hauert Tardit, Hauert Günther Düngerrwerke GmbH, Germany).

### Larval survival and performance assays

To test larval survival and performance, 10 freshly hatched larvae from each subspecies were placed into a 14 cm diameter petri dish containing fresh foliage from one of the focal tree species, with six replicate petri dishes per tree/larval subspecies combination. Foliage was presented in an Eppendorf tube filled with water and sealed with parafilm to avoid desiccation. The number of surviving and dead larvae was recorded every third day, and the surviving larvae were weighed using a Mettler Toledo fine-scale balance (Columbus, OH, USA), we also recorded the instar of the larvae at each point. Larvae were then transferred to a new Petri dish with fresh food. Survival and weight were recorded until all larvae had reached the third instar (i.e., 21 days).

### Ballooning assays

To assess the propensity of early instar larvae to disperse from particular tree species via ballooning, we used five replicates per tree/larval subspecies combination. Each replicate comprised 10 freshly hatched larvae, placed carefully (using a paintbrush) on to leaf material placed in a water-filled Eppendorf tube, which was itself attached to a balsa wood stick (1 cm diameter and 50 cm in length). Each stick was then placed inside a Plexiglas cylinder (20 cm diameter and 1 m in length), with four circular openings covered with mesh to allow airflow (adapted from Capinera & Barbosa, 1976)^50^. The surface of the Eppendorf tube was covered with Vaseline to prevent larvae from crawling down the stick. Filtered air was supplied from the top of the cylinder through a funnel at a constant rate of 0.8 liters per minute (LPM) using a portable pump (Volatile Assay Systems, Renssealer, NY). The number of larvae ballooning (i.e., descending from the leaf surface using silk threads) was recorded during one hour.

### Multiple choice feeding assays

For the multiple-choice feeding assays, individual second-instar larvae were placed at the centre of a 14 cm diameter plastic Petri dish lined with moistened filter paper. Leaf discs (1 cm diameter) from each tree species (or an equivalent area of pine needles) were placed equidistant from one another around the borders of the Petri dish. The initial larval choice was recorded by direct observation during the first hour, and feeding preference was then determined by calculating the total leaf area consumed after 3 and 24 hours. We used 20–22 replicate Petri dishes per larval subspecies. Leaf area consumed was quantified using Adobe Photoshop cc 2015 Software ® (Adobe systems Inc. San Jose, CA, USA). Average leaf area consumed by larvae after 3 and 24 hours was compared using a Friedman test.

### Y-tube olfactometer assays

To assess larval preferences for the volatile blends of the four tree species, we conducted choice tests with individual larvae in a Y-tube olfactometer (1.5 cm inner diameter, 20 cm central tube, 14 cm y-arms), using all pairwise host-tree combinations (oak vs. pine, oak vs. maple, oak vs. beech, pine vs. maple, pine vs. beech, and maple vs. beech). Each of the two arms of the olfactometer was connected to a 10 L cylindrical glass vessel within which 3 g of fresh leaves/needles were placed in a 50 mL glass beaker containing 40 mL water and sealed with parafilm to avoid desiccation. Airflow was controlled by flow meters (Key Instruments, Trevose, PA, USA), with charcoal-filtered air passing over the plant material and entering each arm of the olfactometer at 0.4 L min^−1^.

Each pairwise comparison was performed for 33–42 larvae (replicates) from each subspecies. Fifty second-instar larvae were starved for one day prior to the experiment, and only healthy, non-moulting larvae were used for assays (hence the variation in the number of replicates). The behavioural responses of early instar larvae toward volatiles emitted by the foliage of potential host-trees were recorded with a digital camera (Logitech, Lausanne, Switzerland) for 5 min. A positive response was scored when the larva entered one of the arms of the olfactometer. Larvae that failed to choose any treatment after five minutes were scored as having no preference and excluded from the statistical analyses. Each larva was used only once. Odour position was randomized for each trial, and the olfactometer was cleaned with warm water and unscented soap between trials.

### Plant volatiles

Plant volatiles were collected and analysed as described in Clavijo McCormick *et al*.^62^, using six replicates per tree species from 5gr of fresh leaf material placed in a 50 mL beaker filled with water inside an airtight 10 L glass container, through which charcoal-filtered air was pushed in and pulled out at a flow rate of 1.0 L min^−1^, using a portable volatile-collection unit (Volatile Assay Systems, Rensselaer, New York).

Volatile compounds were trapped using 20 mg Super Q adsorbent filters then eluted with 200 µL dichloromethane containing 1 ng/µL of nonyl acetate as an internal standard. Analyses were performed using a Gas Chromatography-Mass Spectrometry/Flame Ionization Detector (GC-MS/FID) equipped with a 30 m × 250 µm × 0.25 µm DB5-MS column (Wicom GmbH, Heppenheim, Germany). The injector was held at 230 °C, and helium was used as a carrier gas. The GC oven temperature was held at 50 °C for 3 minutes after injection and then increased to 95 °C at a rate of 5 °C/min. Afterwards, the oven was heated to 145 °C with a 15 °C/min gradient, then to 180 °C with a 10 °C/min gradient, and finally to 200 °C with a 10 °C/min gradient. At the end, the oven temperature was held for 3 min at 200 °C. A post-column splitter attached to the MS and FID allowed simultaneous collection of data for tentative identification (MS) and quantification (FID). The resulting data were analysed using the software Mass Hunter and Chemstation (Agilent technologies, Santa Clara, CA US). Volatiles were identified using authentic standards when available, or tentatively identified by comparison with the NIST standard reference database (National Institute of Standards and Technology, Gaithesburg, MD, USA).

## Data Analyses

All data analyses were performed using the statistical software SPSS version 23® (IBM, North Castle, NY, USA). For survival data, we performed a Kaplan-Meier analysis per plant-moth subspecies combination, using a Breslow (Generalised Wilcoxon) test, which emphasizes events occurring at earlier points (i.e. mortality of early instars). We performed two separate analyses, one comparing moth subspecies within the same plant treatment and a second comparing plant species within the moth-subspecies treatment. For weight gain, we calculated the mean weight of freshly molted second instar larvae and used a two-way ANOVA to explore the effects of subspecies, plant, and their interaction on larval weight.

For the ballooning assays, we used an ANOVA followed by a Tukey Post-hoc test. In cases where homoscedasticity requirements were not fulfilled, we opted for a nonparametric Kruskal-Wallis test, followed by Mann-Whitney pairwise comparisons. As before, we performed two separate sets of comparisons, one for tree species within moth subspecies and one for moth subspecies within the same tree species.

For multiple-choice feeding assays, we performed a Chi^2^ test to establish differences in the initial choice made by each larva. For the leaf area consumed after three and twenty-four hours, we used a Friedman test followed by Wilcoxon signed-rank pairwise comparisons. In both cases, the comparisons were conducted for each moth subspecies separately. Due to the nature of the test (dependent samples), no statistical comparisons within plant species were performed.

To analyse differences between paired treatments in the Y-tube assays, we used a Chi^2^ test. We also investigated differences in the volatile emissions of the main classes of chemical compounds (green-leaf volatiles, monoterpenes, sesquiterpenes, and others) between plant species, using ANOVA followed by post-hoc Tukey tests.

## Supplementary information

Supplementary info -Table S1_S2

## Data Availability

The data will be made available upon request.
