# Supplementary material for: Divergent behavioural responses of gypsy moth (Lymantria dispar) caterpillars from three different subspecies to potential host trees
Source: Sci Rep. 2019 Jun 20;9:8953. doi: 10.1038/s41598-019-45201-3 (PMC6586621; doi:10.1038/s41598-019-45201-3)
Supplement: Supplementary file 1 — Supplementary info -Table S1_S2 [file 41598_2019_45201_MOESM1_ESM.pdf]

Supplementary material

Divergent behavioural responses of gypsy moth (*Lymantria dispar*) caterpillars from three different subspecies to potential host trees

Andrea Clavijo McCormick[1,2], Luca Arrigo[2], Helen Eggenberger[2], Mark C. Mescher[2], Consuelo M. De Moraes[2]*

[1] Massey University, Institute of Agriculture and Environment, Private Bag 11222, 4442 Palmerston North, New Zealand

[2] Department of Environmental Systems Science, ETH Zürich, Schmelzbergstarsse 9, 8092 Zürich, Switzerland

*Corresponding author

Correspondence:
E-mail: consuelo.demoraes@usys.ethz.ch
Phone: +41 44 632 39 20
Address: Department of Environmental Systems Science, ETH Zürich, LFO G18, Schmelzbergstarsse 9, 8092 Zürich, Switzerland.

Table S1. Statistical results of a Breslow (Generalized Wilcoxon) test evaluating differences in the survival distribution for each gypsy moth subspecies and host-plant species.

Per gypsy moth subspecies[1]		***Per plant species***	
AGM	Chi^2=2076.158 df=3 P<0.001	*Quercus robur*	Chi^2=570.789 df=2 P<0.001
EGM	Chi^2=1275.56 df=3 P<0.001	*Fagus sylvatica*	Chi^2=106.564 df=2 P<0.001
JGM	Chi^2=551.272 df=3 P<0.001	*Pinus sylvestris*	Chi^2=827.36 df=2 P<0.001
		Acer campestre	Chi^2=25.166 df=2 P<0.001

[1] AGM=Asian gypsy moth, EGM=European gypsy moth, JGM=Japanese gypsy moth.

Table S2. Statistical results of parametric and non-parametric ANOVAs evaluating differences in the percentage of ballooning larvae for each gypsy moth subspecies and host-plant species.

Per gypsy moth subspecies[1,2]		***Per plant species[2]***	
AGM	F=19.78<br>df=3<br>P<0.001	*Quercus robur*	F=10.947<br>df=2<br>P=0.002
EGM	Chi^2=17.085<br>df=3<br>P=0.001	*Fagus sylvatica*	F=5.621<br>df=2<br>P=0.019
JGM	F=11.066<br>df=3<br>P=0.063	*Pinus sylvestris*	F=9.304<br>df=2<br>P=0.004
		Acer campestre	Chi^2=0.385<br>df=2<br>P=0.825

[1] AGM=Asian gypsy moth, EGM=European gypsy moth, JGM=Japanese gypsy moth.

[2] F values correspond to a parametric ANOVA, Chi^2 values to a non-parametric ANOVA (Kruskal-Wallis test).
